# Characterization of *Mariner* transposons in seven species of *Rhus* gall aphids

**DOI:** 10.1038/s41598-021-95843-5

**Published:** 2021-08-11

**Authors:** Aftab Ahmad, Gabriel Luz Wallau, Zhumei Ren

**Affiliations:** 1 School of Life Science, Shanxi University, 92 Wucheng Rd, Taiyuan, 030006 Shanxi China; 2 Departamento de Entomologia e Núcleo de Bioinf Ormática, Instituto Aggeu Magalhães (IAM) - Fundação Oswaldo Cruz (FIOCRUZ), Recife, 50740-465 Brazil

**Keywords:** Phylogenetics, Genomics, Entomology, Phylogeny, Transposition

## Abstract

Transposable elements (TEs), also known as jumping genes, are widely spread in the genomes of insects and play a considerable role in genomic evolution. *Mariner*/DD34D family belongs to class II transposable elements which is widely spread in the genomes of insects and have considerable role in genomic evolution. *Mariner* like elements (MLEs) were searched in the genomes of seven species of *Rhus* gall aphids belonging to six genera. In total, 121 MLEs were detected in the genomes of the seven investigated species of *Rhus* gall aphids, which showed a wide distribution in both close and distant related species. The sequences of MLEs ranged from 1 to 1.4 kb in length and the structural analysis of the MLEs showed that only five copies were potentially active with intact open reading frame (ORF) and terminal inverted repeats (*TIR*s). Phylogenetic analysis showed that all the 121 MLE sequences belonged to four subfamilies, i.e., *Mauritiana*, *Drosophila*, *Vertumana* and *Irritans*, among which *Drosophila* and *Vertumana* subfamilies were reported in aphids for the first time. Our present report revealed the diversity and distribution of MLEs in *Rhus* gall aphid genomes and expanded our understandings on the characterization of transposable elements in aphid genomes, which might be useful as genetic markers and tools and would play an important role in genomic evolution and adaptation of aphids.

## Introduction

Transposable elements (TEs) are DNA sequences (usually less than 15 kb), which have the ability to jump and change its location within the genome, also known as genomic parasites^1,2^. Once these elements exploit the host cellular machinery for their own replication, they may have a large negative impact on the host fitness^1–3^. Transposable elements have considerable influence on the evolution of host genome due to their propagation and replication within host genome^3^. However, very small proportion of TE sequences are currently active with intact open reading frame (ORF) for transposase and most have many deletions and substitution due to vertical inactivation events by the host, and hence they are the inactive remains of once active copies^4^. During transposition, they may disrupt coding or regulatory sequences, and the high similar copies, which dispersed in the genome, can serve as source of non-homologous recombination breaking points resulting in chromosomal rearrangement such as inversion, deletion, translocation, and duplication. Moreover, TEs have the ability to modify the expression of their host genes by juxtaposing new cis-regulatory sequences, and can also be co-opted to new host function and give rise to new host genes^3–6^, through a phenomenon known as molecular domestication^7^. The influence of TEs on genome organization and evolution is not surprising and enough information is available about impact of TEs in the host genome evolution. The complexity and paraphyletic origin of TEs poses substantial challenges to the scientific community, including the detection, classification, assembly, annotations and mapping of genomic variants^8^. Although the recent advancements to the understanding of TE evolution, there are still considerable gaps of knowledge to completely understand the evolutionary interplay between host and genomic parasites^8,9^.

Transposable elements comprised a considerable proportion of eukaryotic and prokaryotic genome^9^, e.g., approximately 3–20% of the genomes in many filamentous fungi^10^, 10%, 12%, 37%, 45% and 80% of the genome in fish, *Caenorhabditis elegans*, mouse, human and some plants, respectively^2,3^. The abundance and widespread distribution of transposable elements required a unified classification to divide these sequences into different lineages though it is still a subject of debate^11–13^. There are many difficulties in classification of TEs, one of which is the analysis of the protein sequences of TEs, because some TEs do not possess any coding sequence while some contain many coding regions with different evolutionary histories due to recombination events^12,13^. Wicker et al. (2007) proposed a unified system to rapidly classify transposable elements, where TEs are classified into two major classes: Class I or retrotransposons (RTs) and Class II or DNA transposons based on their life cycle and molecular structure^11^. The former is transposed by RNA intermediate while DNA transposons are transposed by typical cut and paste mechanism^14^. Based on their sequence compositions and some conserved features, TEs can further be divided into subclasses, orders, superfamilies and families^11–14^.

Class I transposons are divided into two classes: LTR RTs flanked by long terminal repeats (LTRs) and non-LTR RTs with lacking terminal repeats^14^, while Class II elements, or DNA transposons are further classified into two subclasses: subclass 1 elements transpose by the process, i.e., excision and integration, while subclass 2, duplicate before insertion. Among class II TEs in eukaryotes, *Tc1/Mariner* is one of the most abundant superfamily, whose members share many common characteristics^11^. The autonomous copies contain a single ORF, which encodes a transposase of 282 to 350 amino acid residues with the insertion target TA^15,16^. Transposase enzyme has a conserved catalytic triad DDE/D motif and a DNA binding domain containing two helix-turn-helix (HTH) motifs^16^. The major characteristics to distinguish the different *Tc1/Mariner* families are their sequence length and DDE/D signature motif. The length of *Tc1/Mariner* ranges from 1 to 5 kb due to the length of terminal inverted repeats (TIRs) which varies from 13 to 34 bp in *Mariner*, while 20 to 600 bp in *Tc1*. The DDE/D signature motif corresponds to DD34E for *Tc1* and DD34D for *Mariner*^15,16^.

Abundant transposable elements were found in different insect genomes, where the proportion of TEs could also explain the variations of insect genome size^17,18^. So far, insect genome analysis revealed that *Belgica antarctica* had the smallest (99 Mb) genome with TEs less than 1%, while *Locusta migratoria* (6.5 Gb) had the largest one, which consisted of 60% TEs^19^. *Mariner* like elements (MLEs) of *Tc1*/*Mariner* superfamily have a simple structure, including single gene flanked by untranslated sequences and *TIR*s at both 5′ and 3′ ends^20^. *Mariner* transposons were characterized in only a few aphid species in previous studies and very little is known about transposons abundance, diversification and influence on genomic evolution in aphids^4,19–21^. However, many lineages of *Mariner*/DD34D were detected recently in the genomes of three aphid species: *Acyrthosiphon pisum, Diuraphis noxia*, and *Myzus persicae*^19,21^ whose genomes are available at NCBI (http://www.ncbi.nlm.nih.gov/genbank) and in aphid database (http://tools.genouest.org/is/aphidbase), respectively. As, *Mariner* transposons were characterized in only a few aphid species and very little is known about its abundance, diversification and influence on genomic evolution in aphids. In this study, we examined *Mariner* family of *Tc1/Mariner* superfamily of Class II transposons in the genomes of seven species of *Rhus* gall aphids from six genera.

*Rhus* gall aphids (Aphididae: Eriosomatinae: Fordini) include six genera, in which five genera are from east Asia while one from east North America, and specially comprise a unique group^22–25^. *Rhus* gall aphids feed on their primary host plant *Rhus* species (Anacardiceae) to form galls with rich tannins, which were produced as an important medical and industry raw material^22,23^. Recently, Ren et al. investigated the evolutionary relationships within *Rhus* gall aphids by sampling 15 accessions representing all six genera and using 20 gene regions: five nuclear genes as well as 13 protein-coding genes and two rRNA genes of the complete mitochondrial genome, which obtained the backbone phylogeny to well support the monophyly of six genera and resolve the relationship of genera and species from *Rhus* gall aphids^23^.

In case of the seven species in this study, their relationship was as following: the North America genus *Melaphis* was original in East Asia; *Meitanaphis* is sister to *Kaburagia*, and then grouped with *Floraphis*; *Nurudea ibofushi* is nested in *Schlechtendalia* and suggested to be merged in the genus *Schlechtendalia*^22,23^. As transposable elements may serve as genetic markers and tools and have impact on insect genome, adaptation and biology^26^, we are interested in detecting and characterizing *Mariner*/DD34D transposons from at least one *Rhus* gall aphid species from all the six genera, i.e., *Schlechtendalia*, *Nurudea*, *Melaphis*, *Meithanaphis*, *Kaburagia* and *Floraphis* known to feed on *Rhus* species. To our knowledge, this study would represent the first report on the *Mariner* transposable elements and its implications in *Rhus* gall aphids.

## Results

### *Mariner*/DD34D transposons in *Rhus* gall aphids

A survey of the genome projects of seven species of *Rhus* gall aphids (Hemiptera: Aphididae: Eriosomatinae) were carried out for the sequences similar to set of transposase sequences of 50 known MLEs downloaded from the GenBank (see Table 1 in Supplementary File S1). The MLE*s* sequences were used as query in a modified BLASTN search (see “Methods” section for details) against the genome of each studied species to extract homologous sequences. Numbers of hits were identified in the genome of each species against the query sequences. A significant number of hits were predicted to have features that allowed them to be classified in the *M*ariner/DD34D family, while hits corresponded to highly defective elements having no conserved transpose domains were discarded. We also searched the genome of each species for Tc1/DD34E, maT/DD37D, GT/DD39 and VS/DD41D^16^ using consensus sequences of these elements as query, but no good hits (query < 15% and similarity < 30%) were found.

**Figure 1:**
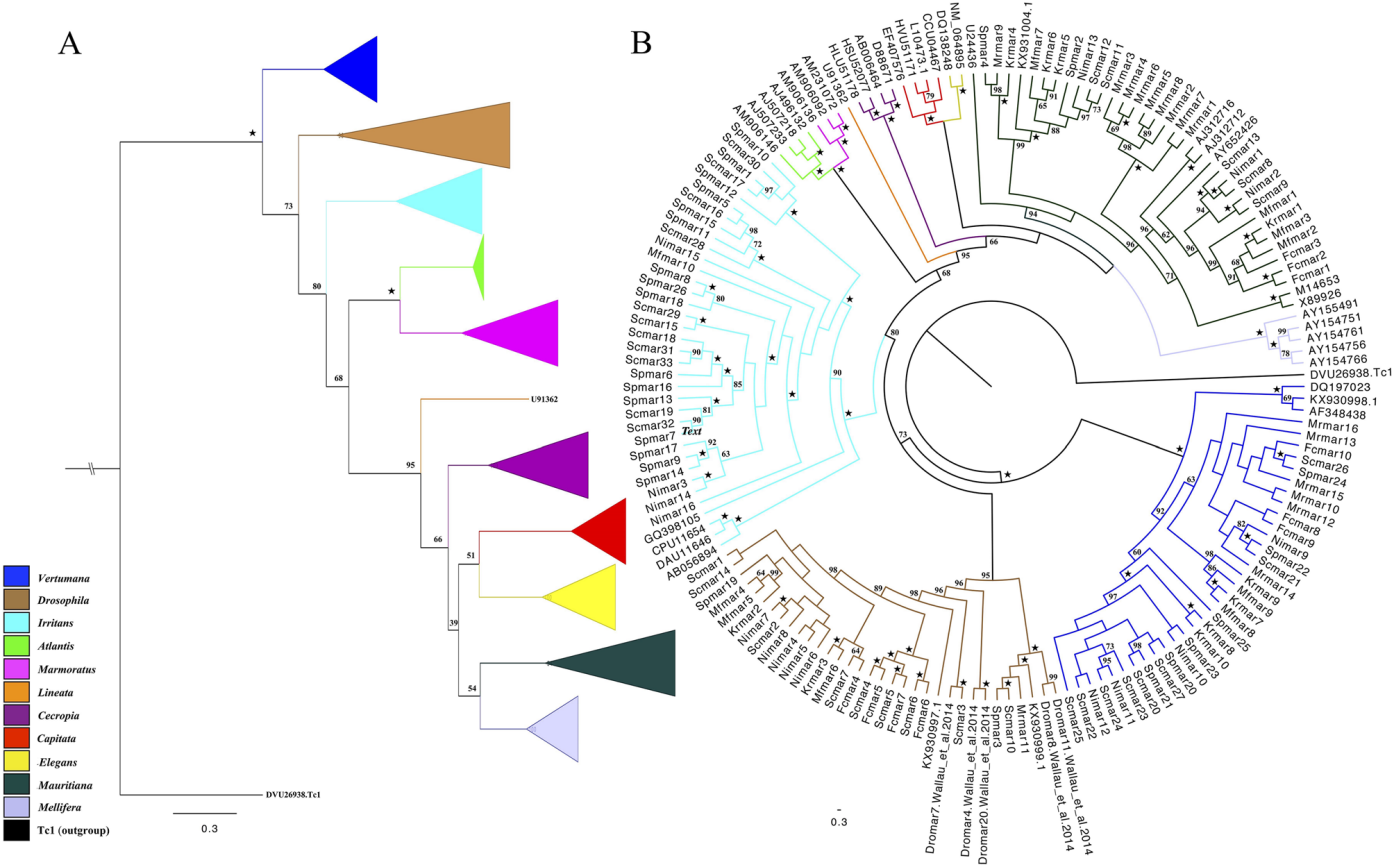
ML phylogenetic tree of *Mariner* lineages constructed with RAxML using GTRGAMMA substitution model. Clade colours denote the different subfamilies of MLEs. **(A)** Different subfamilies of the *Mariner* family with element from *Tc1* family as outgroup. **(B)** 165 *Mariner*-like elements, i.e., 44 from GenBank and 121 from seven species of *Rhus* gall aphids. The branch end showed the NCBI accession numbers of the MLEs downloaded from the database and the names of MLEs detected in this study. *Indicates 100% support by all bootstrap replicates.

We found in total 121 sequences of MLEs in all the seven *Rhus* gall aphid species, i.e., thirty-three in *Schlechtendalia chinensis*, twenty-six in *Schlechtendalia peitan,* ten in *Kaburagia rhusicola, Floraphis choui*, and *Meitanaphis flavogallis,* respectively, and sixteen in *Melaphis rhois* and *Nurudea ibofushi*, respectively. All the detected transposons belonged to different lineages of MLEs, and were classified into four subfamilies of the transposable elements *Mariner*/DD34D family based on the phylogenetic analysis with already classified MLEs from previous studies (see Fig. 1A,B). The numbers and the classifications of MLEs detected in all seven species of *Rhus* gall aphids are shown in Table 1. These results provide the first evidence of the presence of *Mariner*/DD34D transposons in *Rhus* gall aphids.

**Table 1:** The total number of MLEs in all the seven *Rhus* gall aphid species, number of MLEs detected in each species and its classifications into subfamilies of MLEs are shown.

Species	No. of MLEs	MLEs Subfamily				Length (kb)	A/I	TIRs (bp)	C/T
		*Mauritiana*	*Drosophila*	*Vertumana*	*Irritans*				
*Schlechtendalia chinensis*	33	4	9	8	12	1.2–1.4	0/33	22–32	28/5
*Schlechtendalia peitan*	26	2	2	6	16	1.1–1.4	0/26	27–30	23/3
*Nurudea ibofushi*	16	3	5	4	4	1.2–1.4	0/16	25–30	15/1
*Meithanaphis flavogallis*	10	4	3	2	1	1.1–1.4	1/9	27–29	8/2
*Floraphis choui*	10	3	4	3	0	1.1–1.4	1/9	26–30	8/2
*Kaburagia rhusicola*	10	4	2	4	0	1.2–1.4	3/7	26–28	8/2
*Melaphis rhois*	16	9	1	6	0	1.2–1.4	0/16	13–30	16/0

*A/I* potentially active/inactive, *C/T* complete copies/truncated copies.

### General features of *Mariner*/DD34D transposons

The sequences *Mariner*/DD34D transposons detected in this study possessed all the features required for the identification of MLEs. To present the best case scenario of MLEs in *Rhus* gall aphid genomes, we focused on extracting the full length copies, and slightly truncated copies with length 1000 bp or more were also included and reported in the study. MLEs sequences flanked by *TIRs* at both ends were considered full-length. Fifteen of the extracted MLEs were truncated at both or one end among the total 121 detected ones, which were mostly due to their presence at the end of contigs (see Tables 1–4 in Supplementary File S2).

Most of the *Mariner*/DD34D become inactive after invading host genomes by the mechanism called vertical inactivation. The sequences of intact ORF with no stop codon or frameshift mutation can be considered potentially active^19^. Most of the MLEs sequences detected in this study also belonged to inactive lineages either missing intact ORF for transposase or *TIRs*. Only five complete sequences of MLEs with intact ORF and flanked *TIRs* and two truncated copies with complete intact ORF for transposase protein were detected in the study.

Only two (*Scmar7* and *Scmar10*) of the 33 MLEs detected in *Schlechtendalia chinensis* were found to have intact ORF for transposase but truncated with missing *TIRs* at 5′ end. *Kaburagia rhusicola* had ten complete MLEs sequences, of which three (*Krmar2*, *Krmar4* and *Krmar5*) had intact ORF for transposase enzyme and flanked by *TIRs* at both 5′ and 3′ end, and were predicted to be potentially active. Among ten MLEs sequences detected in *Floraphis choui*, only one *(Fcmar4*) has been predicted to be potentially active with intact ORF for transpose and *TIRs* at both ends. While *Meithanaphis flavogllis* had also one potentially active MLE sequence (*Mfmar4*). All the other sequences had at least one or more premature stop codons. No active copy with single intact ORF was detected in *Schlechtendalia peitan, Nurudea ibofushi* and *Melaphis rhois*. Five of the MLEs with intact transposase ORF belonged to the subfamily *Drosophila*, i.e., *Krmar2, Scmar7, Scmar10, Mfmar4* and *Fcmar4*, while two belonged to *Mauritiana*, i.e., *Krmar4* and *Krmar5* (Tables 1–4 in Supplementary File S2). All the MLEs, belonging to *Vertumana* and *Irritans* subfamilies, were inactive with no intact ORF for transposase. All the detected MLEs in the *Rhus* gall aphids have been submitted to GenBank with accession numbers (see Supplementary File S4). In this study we reported the distribution of one hundred-six full length complete and fifteen truncated copies of MLEs in Rhus gall aphids (see Table 1, Supplementary File S4). This study presents the detail view of all the feature of MLEs in *Rhus* gall aphids.

### Structure analysis of *Mariner*/DD34D transposons

*Mariner* like elements (MLEs) are flanked by inverted repeats at their both 5′ and 3′ ends, which can be recognised by transposase enzymes during transposition and are necessary for the mobilization and replications of MLEs in host genomes. Terminal inverted repeats (*TIRs*) were analyzed in all the complete copies of MLEs, and the sequences, belonging to the same subfamily, shared more than 85% similarity. The consensus of *TIRs* in each subfamily are shown in Table 5 in Supplementary File S2). Meanwhile, the TA target site duplication (*TSD*) were also found at both ends in the complete copies except *Krmar8* and *Krmar10*, in which TA was found only at 3′ end. All the completes copies detected were of variable length ranging from 1.2 to 1.35 kb and *TIRs* from 13 to 32 bp (see Table 1, Tables 1–4 in Supplementary File S2).

**Figure 2:**
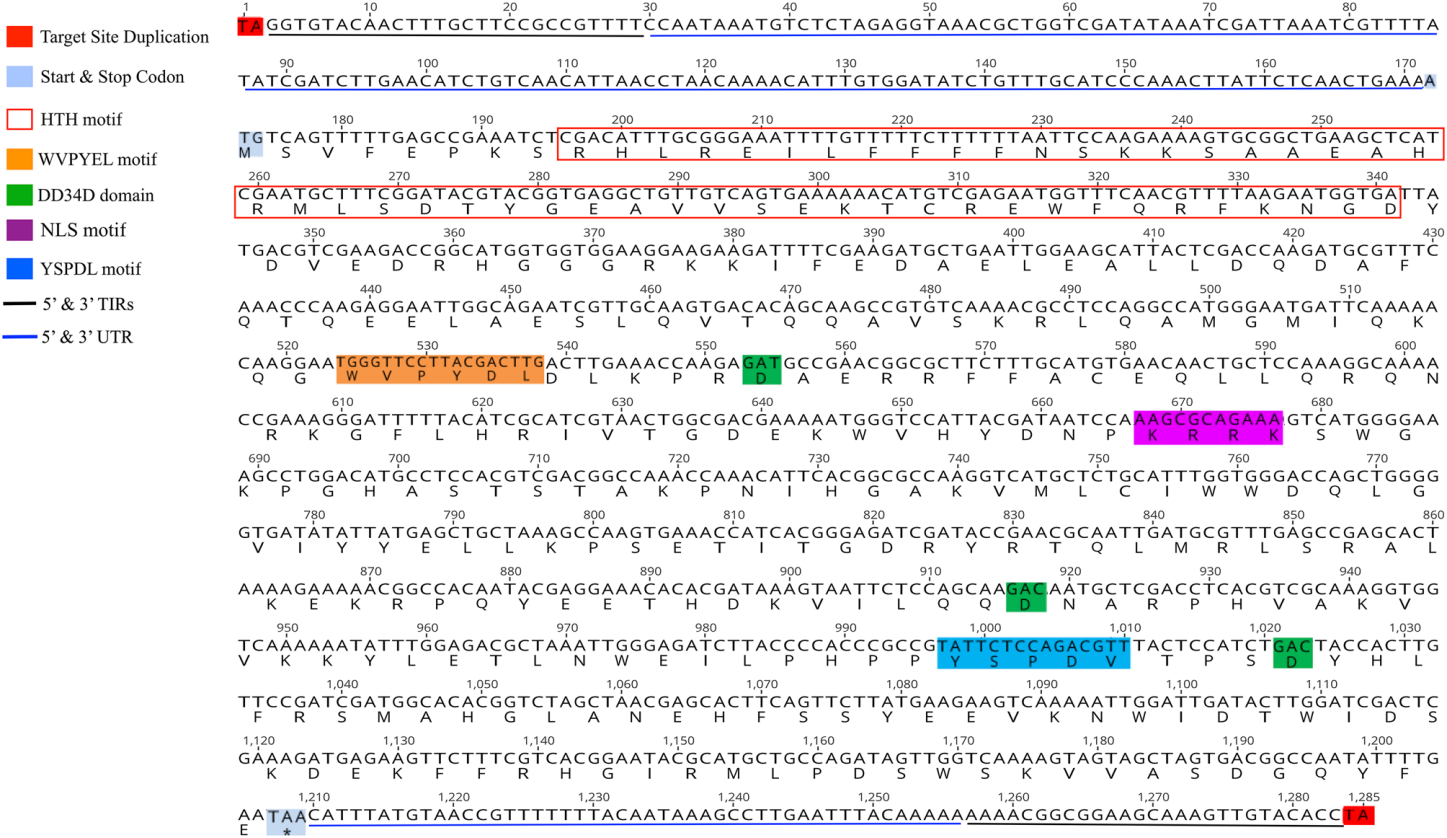
Full length sequence of MLE (*Krmar5*) from *Mauritiana* subfamily in *Kaburagia rhusicola* with slightly modified WVPYE(D)L and YSPDL(V) motifs for transposase.

**Figure 3:**
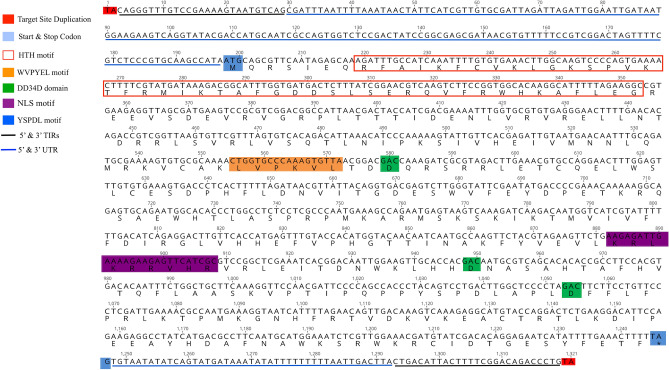
Full length sequence of MLE (*Mfmar4*) from *Drosophila* subfamily in *Meithanaphis flavogallis* with slightly modified W(L)VPY(K)E(V)L motif and NLS for transposase.

**Figure 4:**
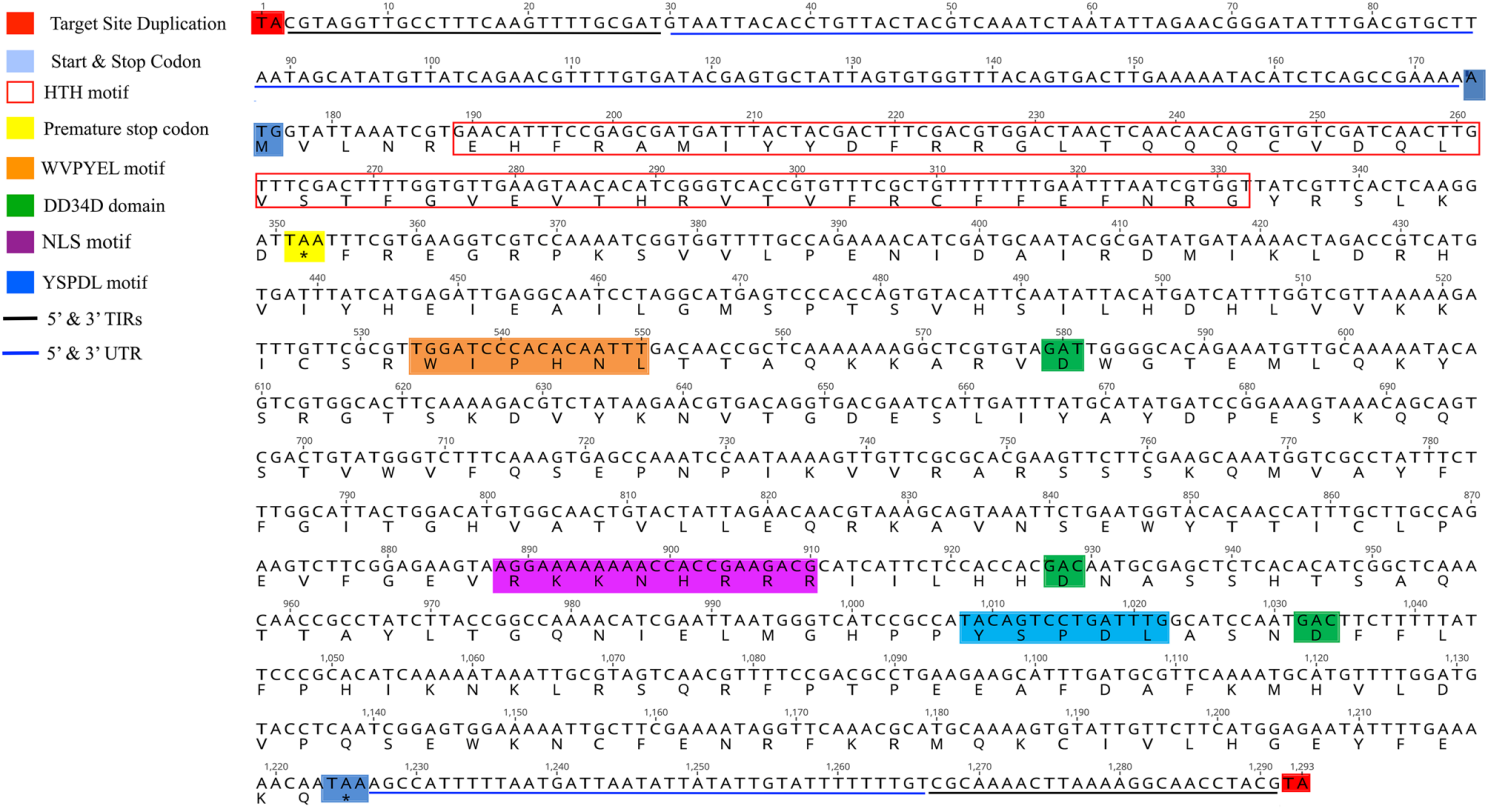
Full length sequence of MLE (*Mrmar18*) from *Vertumana* subfamily in *Melaphis rhois* with a premature stop codon and slightly modified WV(I)PY(H)E(N)L motif and NLS for transposase.

Transposases of the complete MLEs were analyzed for the conserved domains and motifs of *Mariner* transposons. Catalytic domain DD34D were highly conserved in most of the complete copies, while WVPHEL and YSPDL motif required for transposition were slightly modified in some MLEs (see Figs. 2, 3, 4). Helix-turn-helix DNA binding motifs were also conserved and found in all the complete copies. Nuclear localization sequence (NLS) was also present in some complete copies, while absent or modified in others. Some of the detected MLEs became inactive due to presence of only single point mutation (single nucleotide substitution), which led to generate premature stop codon (see Fig. 4). Conserved catalytic domain DD34D, helix-turn-helix (HTH) DNA binding motifs, WVPHEL motif, YSPDL motif and nuclear localization signal (NLS) of three of the complete MLEs, which were detected in this study and belonged to three different subfamilies of MLEs, are shown in Figs. 2, 3 and 4, respectively. The classification and identification of MLEs in *Rhus* gall aphid genomes were further justified by all the conserved domains for transposase.

### Phylogenetic analysis

*Mariner*/DD34D transposons have many common feature and conserved domains which enable them to be placed in a common family (*Mariner*), but have patchy distribution among all the organisms due to differential origin and host speciation events^14,17^. Due to greater variability among the sequences of MLEs, they can be further classified into subfamilies based on sequence similarities among them^14^. The subfamily classification of detected MLEs in *Rhus* gall aphids were done on the basis of DNA sequence similarities. Sequences of well-characterized *Mariner*/DD34D family from other organisms, mainly from the class *insect*, were downloaded from GenBank. However, we did not find any complete and closely related MLEs sequences from other aphids in GenBank. All the downloaded sequences belonged to the reported major subfamilies of *Mariner*/DD34D family, i.e., *Mauritiana*, *Mellifera*, *Irritans*, *Cecropia*, *Capitata*, *Vertumana*, *Drosophila*, *Vertumana*, *Marmoratus*, *Lineata* and *Elegans*.

Phylogenetic relationship of all the 121 MLEs in *Rhus* gall aphids along with the MLE sequences of other organisms, mainly insects, from GenBank were analyzed by constructing ML phylogenetic tree with 1000 duplicates (see “Methods” section for detail). All the detected MLEs of *Rhus* gall aphids were clustered into four subfamilies, i.e., *Mauritiana, Irritans, Vertumana* and *Drosophila*, and they were classified into subfamilies according to their groups and relatedness with the known MLEs from different subfamilies of *Mariner*/DD34D family downloaded from GenBank (see Fig. 1A,B). We identified the MLEs with different lengths and lineages in all seven species of *Rhus* gall aphid, and classified them into respective subfamilies based on their phylogeny.

## Discussion

The seven *Rhus* gall aphid species sampled in this study feed on the primary host plant *Rhus* species and form galls in the leaves of host plant which is rich in tannin, so they have great economic importance to be widely applied in various fields, e.g., medicine, food, dye, chemical and military industry. Like all other aphids, *Rhus* gall aphids are phloem feeding parasites of plants, but unlike other aphids harmful to host plants, they do not damage their host plants, though inducing galls in the leaves of host plant^22,23^. Transposable elements are thought to potentially mediate resistance in insects through changes in gene amplification and mutations in coding sequences, and hence play a role in insect’s genome evolution and adaptations^4^. *Mariner*/DD34D transposons may represent useful genetic tools and provide insights on adaptation and evolution of *Rhus* gall aphids.

We have carried out the first systematic search for *Mariner*/DD34D transposons in the genomic sequences of seven species of *Rhus* gall aphids. The computational search strategy identified 121 MLEs in the genomes of seven species signaling the presence of Class II TEs in the genomes of *Rhus* gall aphids. Our study focused only on the existence of MLEs in *Rhus* gall aphid species, and were irrespective of their total copies, number and percent contribution in the genome. We identified four different subfamilies of *Mariner*/DD34D transposons in our study based on structural and phylogenetic analysis, i.e., *Mauritiana*, *Vertumana*, *Irritans* and *Drosophila* in all the seven species of *Rhus* gall aphids (see Table 1). *Mariner*/DD34D is probably the most widely distributed family of transposons in nature and has been frequently studied, also showed widespread distribution in Hexapods^5,19,27–29^.

A significant amount of *Mariner*/DD34D transposons in the genomes of *Rhus* gall aphids indicated their successful proliferation in the genomes of these aphids. A total of 121 MLEs were detected in all the seven aphid species with variable distribution among the seven species (see Table 1), not following the exact host phylogeny, which was one of the features of transposons^4^. Based on phylogenetic analysis, all the sequences of MLEs were clustered into four subfamilies, among which the subfamily *Vertumana* and *Mauritiana* were the most widely spread ones in all the seven *Rhus* gall aphids, while we identified MLEs from *Vertumana* subfamily and *Drosophila* subfamily in aphid genomes for the first time. MLEs from later two subfamilies were not reported in any other aphid species in previous studies. MLEs from the *Irritans* subfamily were found in three species of *Rhus* gall aphids, i.e., *Schlechtendalia chinensis, Schlechtendalia peitan* and *Nurudea ibofushi*, while absent in the other four studied species (see Table 1). Closely related sequences (based on nucleotide sequence similarities) were clustered into one of the four subfamilies irrespective of their host phylogeny (see Fig. 1B) and percent similarity and distances between all the detected sequences are shown in Supplementary File S5, which predicted the variable distribution of MLEs in *Rhus* gall aphids.

Comparative analysis of all the detected MLEs with the previously reported MLEs in other aphid species, i.e., *Aphis glycine*^4^, *Acyrthosiphon pisum*, *Diuraphis noxia*^19^ and *Acyrthosiphon pisum*^20,21^, also showed patchy distribution as no MLEs from *Mauritiana* were found in the genome mining of *Aphis glycine*^4^, *Diuraphis noxia* and *Acyrthosiphon pisum*^19,21^ while incomplete copies of MLEs from *Mauritiana* subfamily were reported in seven tree aphids^20^. In contrast, MLEs from the subfamily *Irritans* were found in all aphid genomes in previous studies^4,19^ while we have identified MLEs from *Irritans* subfamily in only four species (see Table 1). Partial sequences of *Irritans* and *Mellifera* subfamilies in *Aphis glycine* were identified in vitro by PCR amplification^19,21^, while absence of MLEs from subfamilies like *Mellifera, Capitata* and others in *Rhus* gall aphids might indicate variable distributions of MLEs in aphid genomes, or might be related to the fact that our sequenced genomes didn’t cover the 100% genes and repeat regions of aphid species in the study. MLEs detected in this study was not reported previously in other aphid genomes^4,19–21^, neither we have found any close similarity of these sequences with already reported MLEs in other aphid’s genomes^4,19–21^, which might indicate independent evolution of MLEs from host speciation event.

A fewer of complete copies of each MLE, i.e., 1 to 3 (see Supplementary File S2) was detected in this study as compared to previously proposed studies^5^. Most of the MLEs detected previously in aphids were in vitro by PCR cloning, which resulted in detection of a relatively large number of deleted copies of MLEs^21^. Our study mainly focused on the detection of complete copies of *Mariner*-like elements in *Rhus* gall aphids and very few truncated copies were detected and reported in this study in contrast to previous studies which mainly reported deleted and truncated MLEs mostly less than 1000 bp in aphids^4,19,20^. No Miniature Inverted-repeats transposable elements (MITEs) were detected in this study, which were previously reported in aphids^4,19^.

The relatively low number of different MLEs in aphid genomes in this study as compared to other insects agreed with the previous studies^4,21^, which indicates that the significantly lower distribution of MLEs in aphid genomes might be the special genetic characteristics of the aphids including the *Rhus* gall aphids. Also, this might be due to (i) the genome size sequenced in our study didn’t completely cover the repeated regions in genome due to the sequencing Illumina platform^30^; (ii) around 50–62% of the assembled contigs were < 1000 bp long (see Table 2), which didn’t result in producing good hits by tBLASTn search in the genomes. Though *Tc1/Mariner* is the most abundant superfamily in insect genome, it is poorly represented in aphid genomes^4,5^, which is also supported by our study.

**Table 2:** Species and genomes of all seven *Rhus* gall aphids included in this study, and detailed information of the sequenced genomes of all the species.

Species	Voucher No	Genome size (Mbp)	GC content (%)	No. of contigs	Longest contig (bp)	Shortest contig (bp)	Contigs length ≥ 1000 (%)
*Schlechtendalia chinensis*	Ren A1798	290	34	82,130	607,885	100	38.22
*Schlechtendalia peitan*	Ren A242	277	34.4	250,994	550,865	128	37.37
*Meithanaphis flavogallis*	Ren A2012	235	34.8	169,097	537,951	128	47.73
*Kaburagia rhusicola*	Ren A63	237	35.2	195,776	371,348	128	41.03
*Nurudea ibofushi*	Ren A1796	261	33.8	75,473	297,804	100	48.33
*Floraphis choui*	Ren A403	274	33.7	214,731	616,219	128	43.83
*Melaphis rhois*	Ren A3037	266	34	35,827	624,579	100	44.89

Structural analysis of the protein polypeptides of the detected MLEs in all the seven aphid species showed that the conserved catalytic domains DD34D in the third aspartate residue were mutated in many of the inactive copies, while highly conserved in active copies, which was consistent with the previous studies^31^. DNA binding helix-turn-helix HTH motif and two main conserved domains of MLEs, i.e., WVPHEL and YSPDL, required for transposase activity, which were conserved in most of the MLEs, whereas there was slight modification in the conserved regions in some of the MLEs as shown in Figs. 2, 3 and 4, which were in agreement with the previous finding^20^. Nuclear localization sequence (NLS) motif, being required for the import of transposase to the nucleus, were analyzed and found in some active MLEs (Fig. 2), and were slightly modified in some (Figs. 3, 4) while absent in many sequences due to frequent mutation or inactivation events^32^. However, the previous studies also showed that some of the MLEs didn’t have their own NLS, which depended on other proteins for their nuclear importation^32^.

The current study showed the diversity of MLEs in aphid genomes, but most of the detected MLEs corresponded to inactive lineages, which was in agreement with previous findings^4,18^. The absence of very few potentially active copies supported the phenomenon of vertical inactivation of *Mariner* transposons^5,20^. Single nucleotide substitution which leads to premature stop codon (Fig. 4) and nucleotides loss due to deletions reported in previous studies^2^ appeared to play an important role in vertical inactivation of transposons, e.g., *Irritans* subfamily, had no active MLE copy, i.e., all the copies were inactive with no intact ORF though, they are widely spread in our studied species and in previously studied species^3,18^.

Like all other genes, MLEs are transmitted vertically from parents to offspring during the evolutionary course, so the relationship between MLEs sequences must reflect the evolutionary relationship of their hosts^18,33^. Phylogenetic relationship of aphids based on the mitochondrial genes showed consistency with the classical phylogenetic analysis based on molecular and morphological characteristics in previous studies^33–38^. However, many studies including the recent study of *Tc1/Mariner* TEs in the genomes of nematodes reported significant inconsistency of TEs with their molecular phylogeny as compared to mitochondrial and other single non-transposable genes from the same genome, which indicates MLEs had evolved independently of host speciation event^19,39–44^. We also observed patchy distribution of MLEs in our studied species irrespective of the host phylogenies, which could indicate the independent evolution of MLEs to some degree, also reported by previous studies^45–48^. For instance, MLEs from *Irritans* subfamily were identified in four species but absent in three of the studied species, i.e., *Floraphis choui, Kaburagia rhusicola and Melaphis rhois* (see Table 1), while the distribution of other MLEs among the species were very irregular (see Fig. 1B), irrespective of *Rhus* gall aphid’s phylogeny^22^. We will examine and explain the patchy distribution of these MLEs, and events responsible for this relationship in detail by sampling more species and more MLEs from different subfamilies in our further research.

## Conclusion

This study presented a first report on the diversity and structure composition of *Mariner* transposons of Class II transposable elements in *Rhus* gall aphids. We identified 121 MLEs in seven species of *Rhus* gall aphids which were further classified phylogenetically into four subfamilies: *Mauritiana*, *Drosophila*, *Irritans* and *Vertumana*, among which subfamily *Drosophila* and *Vertumana* were reported for the first time in aphid species. We only demonstrated the presence of full length MLEs including both the active and inactive lineages in *Rhus* gall aphid species and do not find any MLEs reported previously in other aphid species. In our further research, we will examine more TEs and demonstrate the activity of potentially active MLEs, their transposition in *Rhus* gall aphids and role in genome evolution and adaptations of *Rhus* gall aphids as well as the horizontal transfer (HT) of these MLEs in different taxa.

## Methods

All the aphid genomes used in this study were sequenced by shotgun genome skimming method^22,23^, by an already ongoing project in our lab.

### Sample collections

All the mature *Rhus* galls were collected on the host plant grown in natural and wild from different location in China except one species which was collected in North America^22^. There were thousands of aphids in one gall because of the parthenogenetic generations during the gall formation. Some individuals from one gall were placed in 75% alcohol for taxonomic identification using microscopy by following taxonomy protocol^24^. The remaining individuals were preserved in absolute alcohol for DNA extraction. Voucher specimen were deposited at the School of Life Sciences in Shanxi University, China. Sampling information and species taxonomy are shown (see Table 2).

### DNA extraction and sequencing

Three individuals of the aphid samples stored in absolute alcohol were transferred into distilled water for 36 h in 1.5 ml Eppendorf tube, and then the water was removed and the aphids were grounded with the help of a small pestle. Genomic DNA of all samples were extracted using DNeasy extraction kit (QIAGEN, Valencia, CA), and the qualified DNAs were sent to the Genomic Sequencing and Analysis Facility (GSAF), University of Texas, Austin for library construction and next generation sequencing (NGS). A TruSeq Nano DNA library preparation kit (Illumina, FC-121-4003) was used to prepare DNA library and the Illumina NextSeq sequencer was used for the generation of paired-end reads 2 × 150 bp with an insert size of 400 bp. Trimmomatic v.0.35 was used to filter raw data with default settings^49^. De novo assembly of the trimmed data was performed by the program Spades v. 3.7.1^50^ and the whole genome was assembled into contigs with different length. Genome size, GC content and detailed information of the contigs of all the seven *Rhus* gall aphid species were shown in Table 2.

### Data mining

Panel of complete copies of both active and non-active *Mariner* transposable elements were downloaded from GenBank (http://www.ncbi.nlm.nih.gov/GenBank, see Supplementary File S1). Most the downloaded sequences belonged to the class *Insecta*, mainly *Drosophila* and also the MLEs already reported in other species of aphids. Geneious prime 11.0.3 with default parameters (threshold E-value = 10) was used for mining the transposable elements using the downloaded sequences as query in local BLASTn searches on genomic contigs of each species. Detailed information of the sequenced genomes of the seven *Rhus* gall aphid species are given in Table 2. The sequences with the best hits (similarity more than 60% and query coverage more than 60%) were extracted and manually analyzed for MLE signatures and terminal inverted repeats (*TIRs*) following guidelines proposed by previous studies^19^. These threshold values have been set to avoid small sequences which were phylogenetically distant from the *Mariner* family. Each of the complete sequences extracted were used again as query to retrieve more similar sequences following protocol used in previous studies^19^. Truncated sequences with similarity less than 60% and query coverage less than 60% were manually analyzed and were not included and reported in this study due to absence of *TIRs* and any MLEs signatures. No MITEs (Miniature Inverted-repeats Transposable Elements) were retrieved during this study, as per protocol of the previous studies^9^.

### Sequence analysis and identification

All the *Mariner* sequences extracted from each local database of genomic contigs were manually analyzed for its terminal inverted repeats (TIRs) and target site duplications (TSD). Potentially active and non-active copies from the sequences were determined by translating the sequences for transposase using ORF finder implemented in Geneious prime 11.0.3 by default setting. DDD/E catalytic domain and HTH DNA binding conserved domains were analyzed for potentially active and non-active copies of MLEs by NCBI conserved domain search (https://www.ncbi.nlm.nih.gov/Structure/cdd/wrpsb.cgi)^51^ with default parameters, while nuclear localization sequence (NLS) motif for active copies of transposase was searched by cNLS mapper (http://nls-mapper.iab.keio.ac.jp/cgi)^52^. Multiple alignment was done using MAFFT version implemented in Geneious 11.0.3 with default parameters for the analysis of the conserved DDE/D signature in the transposase for potentially active copies.

### ORF and conserved domains of the MLEs

The analysis of the potentially active copies with ORF ranging from 310 to 345 amino acids was performed by aligning them with transposases of *Mariner* family of other organisms downloaded from GenBank. Complete structure composition of the transposable elements, i.e., DNA binding domain (HTH), nuclear localizing motif (NLS) and catalytic domain DD34D of active and inactive copies from each species, was predicted, and the sequences having intact ORF with no stop codon or frameshift mutation were considered active^19^. Conserved catalytic domains DD34D were used to justify the classification of detected TEs into *Mariner* family of *Tc1/Mariner* superfamily. MLEs with no intact ORF and having one or more than one stop codons were also translated and analyzed for conserved domain and motifs, i.e., DD34D catalytic domain, HTH motif, nuclear localization motif, WVPHEL and YSPDL motif. MLEs having no intact ORF and conserved motif and domains due mutations like deletion, insertion or substitution were also classified in the same group based on sequence similarity ≥ 80% in the complete sequence or TIRs as proposed by Wicker et al.

### Phylogenetic analysis

The phylogenetic analysis was performed in order to reveal the relationship of MLEs in *Rhus* gall aphids and other insects. All the sequences detected in *Rhus* gall aphids along with 44 MLEs of other organisms mainly from the class *Insecta* downloaded from GenBank were used to construct the phylogenetic tree (see Supplementary File S3). As not all the detected MLEs had intact ORF in our study, so their whole nucleotide sequences of MLEs were used to align using MAFFT v7.309 multiple alignment implemented in Geneious 11.0.3 with default parameters (Algorithm: FFT-NS-2, Scoring matrix: IPAM/K = 2, Gap open penalty: 1.53, Offset value: 0.123) and construct the phylogram of 121 MLEs detected in *Rhus* gall aphids and other 44 MLEs from GenBank. The Maximum likelihood (ML) phylogenetic tree was constructed by GTRGAMMA model (best fit model) with 1000 replications (bootstraps) using the software RAxML^53^. An MLE from the *Drosophila virilis* (Accession no. DVU26938) belonging to *Tc1* family was used as outgroup.

All the methods performed in this study were in accordance to the relevant rules and guidelines proposed by previous studies^4,8,9,11–14,29,41,45^.

### Ethics approval and consent to participate

All the mature galls and leaves were collected from Wild *Rhus* gall plant species and no permission were required as they were not protected or conserved plant species.

## Supplementary Information

Supplementary Legends.

Supplementary File S1.

Supplementary File S2.

Supplementary File S3.

Supplementary File S4.

Supplementary File S5.

## Data Availability

The genomic data (sequenced genomes) of *Rhus* gall aphid species used and generated in this study are not publicly available yet and deposited in the School of life Sciences, Shanxi University under voucher numbers mentioned in the manuscript. The genomic data used in this study can also be provided to readers by the author(s) upon request. All the other data generated in this study, i.e., *Mariner*/DD34D transposons used as queries, MLEs used for phylogenetic analysis and detected MLEs are included in this article and its Supplementary Files.
