# Supplementary material for: Characterization of Mariner transposons in seven species of Rhus gall aphids
Source: Sci Rep. 2021 Aug 11;11:16349. doi: 10.1038/s41598-021-95843-5 (PMC8357937; doi:10.1038/s41598-021-95843-5)
Supplement: Supplementary file 1 — Supplementary Legends. [file 41598_2021_95843_MOESM1_ESM.pdf]

Supplementary Data:

Supplementary file S1:

Table 1. MLEs of different organisms used as queries in tBLASTn along with their accession numbers and their subfamilies classification.

Supplementary file S2:

Table 1. Detailed information with GenBank accession number of each MLE from *Mauritiana* subfamily detected in all *Rhus* gall aphid species, i.e., *Schlechtendalia chinensis, Schlechtendalia peitan, Nurudea ibofushi, Meithanphis flavogallis, Floraphis choui, Kaburagia rhusicola, Melaphis rhois.*

Table 2. Detailed information with GenBank accession number of each MLE from *Drosophila* subfamily detected in all seven *Rhus* gall aphid species.

Table 3. Detailed information with GenBank accession number of each MLE from *Vertumana* subfamily detected in all seven *Rhus* gall aphid species.

Table 4. Detailed information with GenBank accession number of each MLE from *Irritans* subfamily detected in three *Rhus* gall aphid species, i.e., *Schlechtendalia chinensis*, *Schlechtendalia peitan*, *Nurudea ibofushi*.

Table 5. Consensus sequences of 5' TIRs and 3' TIRs of the detected MLEs belonging to four subfamilies of *mariner* family in seven species of *Rhus* gall aphids.

Supplementary file S3:

Fasta file containing all the 44 MLEs sequences of 11 subfamilies from GenBank used in the construction of ML tree.

Supplementary file S4:

Fasta file containing all the MLEs sequences detected in all the seven species of *Rhus* gall aphids along with GenBank accession numbers.

Supplementary file S5:

Spread sheet (csv) showing percent similarities and distances among all the detected MLEs sequences in seven species of *Rhus* gall aphids and MLEs from GenBank used as Query.

Note: Only homologous sequences from GenBank are shown in this file and query sequences which did not result any good hit during blast search are no included.
