# Supplementary material for: Characterization of Mariner transposons in seven species of Rhus gall aphids
Source: Sci Rep. 2021 Aug 11;11:16349. doi: 10.1038/s41598-021-95843-5 (PMC8357937; doi:10.1038/s41598-021-95843-5)
Supplement: Supplementary file 2 — Supplementary File S1. [file 41598_2021_95843_MOESM2_ESM.docx]

**Table 1.** List of MLEs of different organisms used as queries in tBLASTn along with there Accession number and information of Subfamily of MLEs they belong.

| Accession No. | Species name | MLE name | MLE Subfamily |
| --- | --- | --- | --- |
| KX9309994 | Bactrocera tryoni | Batmar6 | Drosophila |
| KX930997 | ″ | Batmar9 | Drosophila |
| KX930998 |  | Batmar13 | Vertumana |
| KX930999 |  | Batmar14 | Drosophila |
| KX931004 |  | Batmar11 | Mauritiana |
| M14653 | Drosophila Mauritiana | Dromar | Mauritiana |
| Wallau et al. 2014 | Drosophila melanogester | Dromar7 | Drosophila |
| Wallau et al. 2014 | Drosophila erecta | Dromar8 | ″ |
| Wallau et al. 2014 | Drosophila simmulans | Dromar11 | ″ |
| Wallau et al. 2014 | Drosophila ananassae | Dromar28 | ″ |
| X78906 | Drosophila Mauritiana | - | Mauritiana |
| AF035566 | Drosophila teissieri | DtBz3 | Mauritiana |
| AB006464 | Attacus atlas | Aamar | Cecropia |
| AC006747 | Caenorhabditis elegans | Cemar | Elegans |
| AF348438 | Bactrocera neohumeralis | Bnmar | Vertumana |
| AJ312712 | Bombus terrestris | Botmar1-1 | Mauritiana |
| AJ312712 | Bombus terrestris | Botmar1-5 | Mauritiana |
| AJ496130 | Alvinella caudata | Alvcmar1.11 | Atlantis |
| AJ496131 | ″ | Alvcmar1.12 | Atlantis |
| AJ496132 | ″ | Alvcmar1.13 | Atlantis |
| AM906136 | Portunus pelagicus | Porpmar1 | Vertumana |
| AY154747 | Chymomyza amoena | Camar1.1 | Mellifera |
| AY154750 | ″ | Camar1.7 | Mellifera |
| AY154756 | Epicauta funebris | Efmar1.1 | Mellifera |
| AY154761 | Forficula auricularia | Famar1.1 | Mellifera |
| AY154766 | Ceratitis capitata | Ccmar2.1 | Mellifera |
| AY155491 | Chymomyza amoena | Camar1 | Mellifera |
| AY282463 | Trichomonas vaginalis | Tvmar1 | Marmoratus |
| AY652426 | Myrmica ruginodis | Myrmar2-4 | Mauritiana |
| CPU11654 | Chrysoperla plorabunda | Cpmar8 | Irritans |
| D88671 | Bombyx mori | Bmmar | Cecropia |
| DAU11646 | Drosophila ananassae | Damar14 | Irritans |
| DQ197023 | Teleopsis quinqueguttata | Tqmar1.1 | Vertumana |
| GQ398105 | Agrilus planipennis | Apmar1 | Irritans |
| HVU51171 | Hydra vulgaris | Hvmar1 | Capitata |
| HVU1183 | Hydra vulgaris | Hvmar | Capitata |
| PPU04452 | Phlebotomus papatasii | Ppmar24.2 | Cecropia |
| U91362 | Nabis sp. HMR-1997a | Dbmar4 | Lineata |
| X71979 | Girardia tigrina | Dtmar1 | Cecropia |
| X73312 | Myrmica ruginodis | Myrmar | Mauritiana |
| X89926 | Drosophila Mauritiana | Dromar c351 | Mauritiana |
| GQ231493 | Aphis glycine | Agmar1 | Irritans |
| GQ231494 | Aphis glycine | Agmar2 | Mellifera |
| AB858399 | Aphis gossypii | Agos1.1 | Mauritiana |
| AB858404 | Aphis pomi | Apom1.1 | Mauritiana |
| AB858409 | Aphis spiraecola | Aspimar 1.1 | Mauritiana |
| AB858417 | Hyalopterus pruni | Hprumar 1.1 | Mauritiana |
| AB858430 | Toxoptera aurantii | Taurmar 1.1 | Mauritiana |
| NM_064895 | Caenorhabditis elegans | T12B5.6 | Elegans |
