# Supplementary material for: Characterization of Mariner transposons in seven species of Rhus gall aphids
Source: Sci Rep. 2021 Aug 11;11:16349. doi: 10.1038/s41598-021-95843-5 (PMC8357937; doi:10.1038/s41598-021-95843-5)
Supplement: Supplementary file 3 — Supplementary File S2. [file 41598_2021_95843_MOESM3_ESM.docx]

**Table 1.** Detected MLEs from *Mauritiana* subfamily in *Rhus* gall aphids species (*Schlechtendalia chinensis, Schlechtendalia peitan, Nurudea ibofushi, Meithanphis flavogallis, Floraphis choui, Kaburagia rhusicola, Melaphis rhois*).

| Accession No. | Lineage name | Complete copy number | Length (bp) | TIR length | Intact ORF for transposase |
| --- | --- | --- | --- | --- | --- |
| MZ489043 | Scmar8 | 1 | 1276 | 29 | No |
| MZ489044 | Scmar9 | 1 | 1259 | 29 | No |
| MZ489046 | Scmar11 | 1 | 1283 | 29 | No |
| MZ489047 | Scmar12 | 1 | 1237 | 29 | No |
| MZ489048 | Scmar13 | 1 | 1271 | 22 | No |
| MZ489070 | Spmar2 | 1 | 1284 | 29 | No |
| MZ489072 | Spmar4 | 1 | 1227 | 29 | No |
| MZ489020 | Nimar1 | 1 | 1276 | 27 | No |
| MZ489021 | Nimar2 | 1 | 1259 | 26 | No |
| MZ489032 | Nimar13 | 1 | 1238 | 27 | No |
| MZ488994 | Mfmar1 | 1 | 1312 | 28 | No |
| MZ488995 | Mfmar2 | 1 | 1299 | 28 | No |
| MZ488996 | Mfmar3 | 1 | 1263 | 28 | No |
| MZ489000 | Mfmar7 | 0 | 1158 | Truncated ends | No |
| MZ488975 | Fcmar1 | 1 | 1289 | 29 | No |
| MW699035 | Fcmar2 | 1 | 1283 | 28 | No |
| MZ488976 | Fcmar3 | 0 | 1152 | Truncated ends | No |
| MZ488984 | Krmar1 | 1 | 1247 | 23 | No |
| MZ488987 | Krmar4 | 1 | 1286 | 28 | Yes |
| MZ488988 | Krmar5 | 1 | 1285 | 27 | Yes |
| MZ4889849 | Krmar6 | 1 | 1269 | 27 | No |
| MZ489004 | Mrmar1 | 1 | 1261 | 28 | No |
| MZ489005 | Mrmar2 | 1 | 1260 | 29 | No |
| MZ489006 | Mrmar3 | 1 | 1259 | 29 | No |
| MZ489007 | Mrmar4 | 1 | 1256 | 29 | No |
| MZ4890048 | Mrmar5 | 1 | 1259 | 29 | No |
| MZ489009 | Mrmar6 | 1 | 1247 | 29 | No |
| MZ489010 | Mrmar7 | 1 | 1276 | 21 | No |
| MZ489011 | Mrmar8 | 1 | 1212 | 13 | No |
| MZ489012 | Mrmar9 | 1 | 1267 | 29 | No |

| Accession No. | Lineage name | Complete copy number | Length (bp) | TIR length | Intact ORF for transposase |
| --- | --- | --- | --- | --- | --- |
| MZ489036 | Scmar1 | 1 | 1316 | 28 | No |
| MZ489037 | Scmar2 | 1 | 1316 | 28 | No |
| MZ489038 | Scmar3 | 0 | 1264 | Truncated ends | No |
| MZ489039 | Scmar4 | 1 | 1322 | 30 | No |
| MZ489040 | Scmar5 | 1 | 1311 | 30 | No |
| MZ489041 | Scmar6 | 0 | 1273 | Truncated 5' TIR | No |
| MZ489042 | Scmar7 | 0 | 1132 | Truncated ends | Yes |
| MZ489045 | Scmar10 | 0 | 1188 | Truncated 5' TIR | Yes |
| MZ489049 | Scmar14 | 1 | 1315 | 31 | No |
| MZ489071 | Spmar3 | 1 | 1272 | 27 | No |
| MZ489076 | Spmar8 | 1 | 1275 | 29 | No |
| MZ489023 | Nimar4 | 0 | 1291 | Truncated 5' TIR | No |
| MZ489024 | Nimar5 | 1 | 1288 | 26 | No |
| MZ489025 | Nimar6 | 1 | 1351 | 28 | No |
| MZ489026 | Nimar7 | 1 | 1316 | 26 | No |
| MZ489027 | Nimar8 | 1 | 1310 | 26 | No |
| MZ489097 | Mfmar4 | 1 | 1321 | 27 | Yes |
| MZ489098 | Mfmar5 | 1 | 1319 | 27 | No |
| MZ489099 | Mfmar6 | 1 | 1275 | 27 | No |
| MZ488977 | Fcmar4 | 1 | 1298 | 25 | Yes |
| MZ488978 | Fcmar5 | 1 | 1322 | 30 | No |
| MZ488979 | Fcmar6 | 0 | 1276 | Truncated 5' TIR | No |
| MZ488980 | Fcmar7 | 1 | 1312 | 30 | No |
| MZ488985 | Krmar2 | 1 | 1324 | 27 | Yes |
| MZ488986 | Krmar3 | 1 | 1279 | 28 | No |
| MZ489014 | Mrmar11 | 1 | 1309 | 28 | No |

**Table 2.** Detected MLEs from *Drosophila* Subfamily in *Rhus* gall aphids species (*Schlechtendalia chinensis, Schlechtendalia peitan, Nurudea ibofushi, Meithanaphis flavogallis, Floraphis choui, Kaburagia rhusicola, Melaphis rhois*).

**Table 3.** Detected MLEs from *Vertumana* Subfamily in *Rhus* gall aphids species (*Schlechtendalia chinensis, Schlechtendalia peitan, Nurudea ibofushi, Meithanaphis flavogalis, Floraphis choui, Kaburagia rhusicola, Melaphis rhois*)

| Accession No. | Lineage name | Complete copy number | Length (bp) | TIR length | Intact ORF for transposase |
| --- | --- | --- | --- | --- | --- |
| MZ489055 | Scmar20 | 1 | 1295 | 27 | No |
| MZ489056 | Scmar21 | 1 | 1273 | 27 | No |
| MZ489057 | Scmar22 | 1 | 1287 | 27 | No |
| MZ489058 | Scmar23 | 2 | 1287 | 29 | No |
| MZ489059 | Scmar24 | 1 | 1275 | 27 | No |
| MZ489060 | Scmar25 | 1 | 1291 | 27 | No |
| MZ489061 | Scmar26 | 2 | 1252 | 25 | No |
| MZ489062 | Scmar27 | 1 | 1290 | 27 | No |
| MZ489088 | Spmar20 | 1 | 1281 | 29 | No |
| MZ489089 | Spmar21 | 1 | 1293 | 29 | No |
| MZ489090 | Spmar22 | 1 | 1263 | 28 | No |
| MZ489091 | Spmar23 | 1 | 1247 | 26 | No |
| MZ489092 | Spmar24 | 1 | 1252 | 27 | No |
| MZ489093 | Spmar25 | 1 | 1281 | 27 | No |
| MZ489028 | Nimar9 | 1 | 1305 | 28 | No |
| MZ489029 | Nimar10 | 1 | 1286 | 25 | No |
| MZ489030 | Nimar11 | 1 | 1291 | 26 | No |
| MZ489031 | Nimar12 | 1 | 1295 | 25 | No |
| MZ489001 | Mfmar8 | 1 | 1245 | 24 | No |
| MZ489002 | Mfmar9 | 2 | 1196 | 23 | No |
| MZ488981 | Fcmar8 | 1 | 1287 | 26 | No |
| MZ488982 | Fcmar9 | 2 | 1268 | 27 | No |
| MZ488990 | Krmar7 | 1 | 1285 | 27 | No |
| MZ488991 | Krmar8 | 1 | 1265 | 22 | No |
| MZ488992 | Krmar9 | 0 | 1188 | Truncated end | No |
| MZ488993 | Krmar10 | 0 | 1244 | Truncated end | No |
| MZ489013 | Mrmar10 | 1 | 1288 | 23 | No |
| MZ489015 | Mrmar12 | 1 | 1272 | 26 | No |
| MZ489016 | Mrmar13 | 1 | 1289 | 26 | No |
| MZ489017 | Mrmar14 | 1 | 1318 | 26 | No |
| MZ489018 | Mrmar15 | 1 | 1296 | 26 | No |
| MZ489019 | Mrmar16 | 1 | 1293 | 26 | No |

**Table 4.** Detected MLEs from Irritans Subfamily in *Rhus* gall aphids species (*Schlechtendalia chinensis, Schlechtendalia peitan, Nurudea ibofushi).*

| Accession No. | Lineage name | Complete copy number | Length (bp) | TIR length | Intact ORF for transposase |
| --- | --- | --- | --- | --- | --- |
| MZ489050 | Scmar15 | 1 | 1297 | 27 | No |
| MZ489051 | Scmar16 | 1 | 1291 | 28 | No |
| MZ489052 | Scmar17 | 1 | 1241 | 30 | No |
| MZ489053 | Scmar18 | 1 | 1260 | 25 | No |
| MZ489054 | Scmar19 | 1 | 1291 | 23 | No |
| MZ489063 | Scmar28 | 1 | 1289 | 30 | No |
| MZ489064 | Scmar29 | 1 | 1289 | 28 | No |
| MZ489065 | Scmar30 | 1 | 1231 | 28 | No |
| MZ489066 | Scmar31 | 0 | 1184 | truncated | No |
| MZ489067 | Scmar32 | 1 | 1284 | 24 | No |
| MZ489068 | Scmar33 | 1 | 1413 | 29 | No |
| MZ489069 | Spmar1 | 1 | 1294 | 30 | No |
| MZ489073 | Spmar5 | 1 | 1291 | 30 | No |
| MZ489074 | Spmar6 | 0 | 1196 | Truncated 5' TIR | No |
| MZ489075 | Spmar7 | 0 | 1217 | Truncated 5' TIR | No |
| MZ489076 | Spmar8 | 1 | 1287 | 28 | No |
| MZ489077 | Spmar9 | 1 | 1281 | 30 | No |
| MZ489078 | Spmar10 | 1 | 1232 | 28 | No |
| MZ489079 | Spmar11 | 1 | 1288 | 30 | No |
| MZ489080 | Spmar12 | 1 | 1235 | 28 | No |
| MZ489081 | Spmar13 | 1 | 1284 | 28 | No |
| MZ489082 | Spmar14 | 1 | 1279 | 28 | No |
| MZ489083 | Spmar15 | 1 | 1279 | 28 | No |
| MZ489084 | Spmar16 | 1 | 1249 | 28 | No |
| MZ489085 | Spmar17 | 1 | 1279 | 28 | No |
| MZ489086 | Spmar18 | 1 | 1286 | 24 | No |
| MZ489094 | Spmar26 | 1 | 1285 | 28 | No |
| MZ489022 | Nimar3 | 1 | 1284 | 28 | No |
| MZ489033 | Nimar14 | 1 | 1272 | 28 | No |
| MZ489034 | Nimar15 | 1 | 1232 | 28 | No |
| MZ489035 | Nimar16 | 1 | 1276 | 27 | No |
| MZ489003 | Mfmar10 | 0 | 1215 | 3' truncated | No |

**Table 5.** Consensus sequences of 5' TIRs and 3' TIRs of the detected MLEs from all four subfamilies in this study.

| MLE subfamily | 5' TIRs | 3' TIRs |
| --- | --- | --- |
| *Mauritiana* | TATCAGGTGTATAAATTTGAATCCGCAGTTT | ATAGTCCACATATTTAAACTTWGGCGTCAAA |
| *Drosophila* | TACAGGGTTTGTCCGAAAAGTAATGTCAGT | ATGTCCCAAACAGGCTTTTCATTACAGTCA |
| *Vertumana* | TATACGTAGGTTGCCTTTTAAGTTTTGCGA | ATATGCATCCAACGGAAAATTCAAAACGCT |
| *Irritans* | TATACGAGRGTCGGCTGAAAAGTCCCGGGCCT | ATATGCTCCCAGCCGACTTTTCAAGGTCCGGA |

Note: The highlighted nucleotide in the consensus sequences shows the mismatches.
